# Bis{μ_4_-*N*-[phen­yl(pyridin-2-yl­aza­nid­yl)meth­yl]pyridin-2-aminido}tetra­kis(tetra­hydro­furan)­tetra­lithium

**DOI:** 10.1107/S1600536813031838

**Published:** 2013-11-27

**Authors:** Juan Chen

**Affiliations:** a Department of Chemistry, Taiyuan Teachers College, Taiyuan 030031, People’s Republic of China

## Abstract

The title complex, [Li_4_(C_17_H_14_N_4_)_2_(C_4_H_8_O)_4_], bears a novel tetra­dentate di­amido ligand. In the tetra­nuclear centrosymmetric complex mol­ecule, the metal atoms exhibit two kinds of coordination modes. The middle two Li^+^ cations are coord­inated by four N (ligand) and one O (tetra­hydro­furan, THF) atoms, resulting in a distorted square-pyramidal geometry. The outer two Li^+^ cations are in distorted tetra­hedral environments consisting of three N (ligand) and one O (THF) atoms. The Li—N bond lengths vary from 2.020 (7) to 2.441 (6)Å.

## Related literature

For reviews of related metal amides, see: Holm *et al.* (1996 ▶); Kempe (2000 ▶). For reviews of amidinates, see: Edelmann (1994 ▶); Mohamed (2010 ▶). For related organometallic compounds with amino­pyridinato ligands, see: Kempe (2003 ▶); Smolensky *et al.* (2005 ▶); Talja *et al.* (2008 ▶); Polamo & Leskela (1996 ▶).
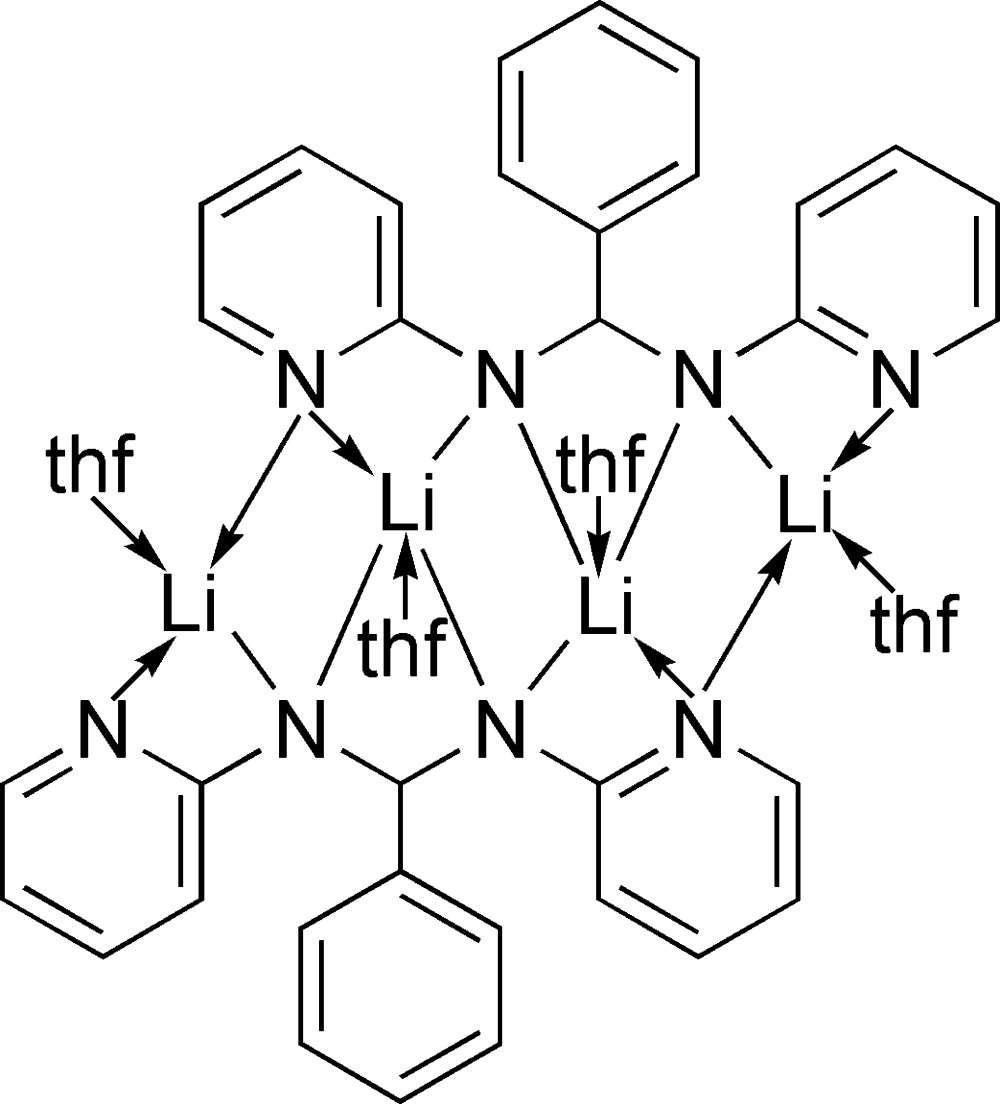


## Experimental

### 

#### Crystal data

[Li_4_(C_17_H_14_N_4_)_2_(C_4_H_8_O)_4_]
*M* *_r_* = 864.82
Triclinic, 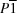

*a* = 10.3322 (10) Å
*b* = 11.2231 (11) Å
*c* = 12.4813 (12) Å
α = 111.021 (2)°
β = 105.355 (2)°
γ = 100.763 (2)°
*V* = 1237.3 (2) Å^3^
*Z* = 1
Mo *K*α radiation
μ = 0.07 mm^−1^
*T* = 295 K
0.20 × 0.15 × 0.15 mm

#### Data collection

Bruker SMART CCD diffractometer
Absorption correction: multi-scan (*SADABS*; Sheldrick, 1996 ▶) *T*_min_ = 0.986, *T*_max_ = 0.989
6796 measured reflections
4339 independent reflections
2180 reflections with *I* > 2σ(*I*)
*R*_int_ = 0.036

#### Refinement

*R*[*F* ^2^ > 2σ(*F* ^2^)] = 0.073
*wR*(*F* ^2^) = 0.243
*S* = 0.93
4339 reflections
298 parameters
61 restraints
H-atom parameters constrained
Δρ_max_ = 0.37 e Å^−3^
Δρ_min_ = −0.24 e Å^−3^

### 

Data collection: *SMART* (Bruker, 2000 ▶); cell refinement: *SAINT* (Bruker, 2000 ▶); data reduction: *SAINT*; program(s) used to solve structure: *SHELXS97* (Sheldrick, 2008 ▶); program(s) used to refine structure: *SHELXL97* (Sheldrick, 2008 ▶); molecular graphics: *SHELXTL* (Sheldrick, 2008 ▶); software used to prepare material for publication: *SHELXS97*.

## Supplementary Material

Crystal structure: contains datablock(s) I, New_Global_Publ_Block. DOI: 10.1107/S1600536813031838/rk2418sup1.cif

Structure factors: contains datablock(s) I. DOI: 10.1107/S1600536813031838/rk2418Isup2.hkl

Additional supplementary materials:  crystallographic information; 3D view; checkCIF report

## Figures and Tables

**Fig. 1.**
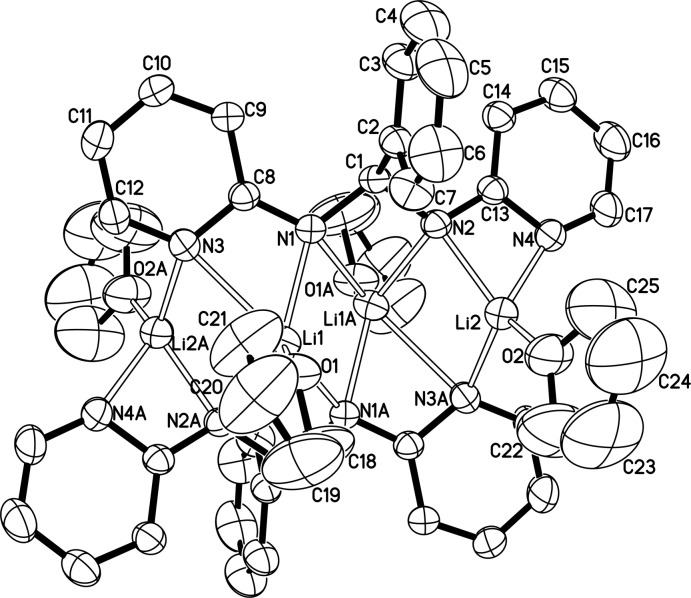
The molecular structure, showing the atom-numbering scheme. Displacement ellipsoids are drawn at the 30% probability level. H atoms are omitted for clarity.

## supplementary crystallographic information

### 1. Comment

The exploration of ancillary ligand systems supporting catalytically active metal centers is a long-standing demand in the coordination chemistry. The *N*-donor ligands are important alterneatives instead of the ubiquitous cyclopentadienyl species. Metal amides were found having valuable applications in various industrial and biological processes (Holm *et al.*, 1996; Kempe, 2000). Amidinate ligands have been extensively studied for decades due to their high adaptability to a wide variety of metals and the remarkable utility as homogeneous catalysts for olefin polymerization of corresponding metal complexes (Edelmann, 1994; Mohamed, 2010). As the closest "relatives" amidinates, pyridyl amido ligands like [N(*R*)(*PY*)]^-^ with flexible bonding modes such as the strained *N,N'*-chelating fashion and the bimetallic bridging binding fashion, have attracted much attention (Kempe, 2003; Smolensky *et al.*, 2005; Talja *et al.*, 2008). Recently, a special *N*-functionalized aminopyridinato ligand was developed by introducing a linker between two aminopyridinato moieties, possessing the *η^3^*:*η^3^* environment. Here, the synthesis and crystal structure of a new lithium complex supported by this ligand will be described.

Aminal bis(2-pyridylamino)toluene is the precursor of the title compound. It was prepared by condensation of two equivalents of 2-aminopyridine and one equivalent of benzaldehyde *via* reflux in methanol. After lithiation of bis(2-pyridylamino)toluene with two equivalents of butyllithium in diethyl ether, it gave yellow crystals of diamide derivative·However, the crystalline qualitity was not good enough for X-ray crystallography analysis. It is solvated with one molar of *Et*_2_O donor inferred from its ^1^H NMR spectrum. The suitable for X-ray investigation single-crystal of the title compound was obtained by recrystallization in *THF*. Its molecular structure is shown in Fig. 1. It is revealed as a tetranuclear species. The metal centers are bound in a zone composed by two tetradentate diamido ligands and they are seprerated in a zigzag mode with distances of 2.578 (10)Å and 2.633 (8)Å. The Li^···^Li distances are obviously longer than that of 2.399 (12)Å in the reported {[NH(*Ph*)(2-C_5_H_4_N)]Li[N(*Ph*)(2-C_5_H_4_N)]}_2_ (Polamo & Leskela, 1996). Each Li is covered by a *THF* molecule from the outer direction. The molecule is centrosymmetric and its center of inversion coincide with the central point of the middle [LiN]_2_ core. There are two different coordination environments employed towards lithium atoms. The middle two lithium atoms are coordinated with four N and one O atoms, resulting in the five-coordinate distorted quadrangular pyramidal geometry. The outer two lithium atoms are in the distorted tetrahedral environment consisting of three N and one O atoms. The distances of Li–N bonds are varying from 2.02 to 2.44Å.

### 2. Experimental

A solution of *n*-*Bu*Li (1.6 *M*, 2.4 ml, 3.8 mmol) in hexane was slowly added into a solution of di(2-pyridylamino)toluene {*Ph*CH[(2-C_5_H_4_N)NH]_2_} (0.53 g, 1.9 mmol) in *Et*_2_O (30 ml) at 273 K by syringe. The mixture was stirred at room temperature for five hours. Then all the volatiles were removed under vacuum. The residue was recrystallized with *THF* (20 ml), it gave the title compound as yellow crystals (yield 0.52 g, 62%). M.p.: 413-415 K. ^1^H NMR (300 MHz, C_6_D_6_) δ: 8.3-5.5 (m, 13H;*Ph* and pyridyl), 3.260 (s, 8H; O–CH_2_ of *THF*), 1.212 (s, 8H; C–CH_2_ of *THF*); ^13^C NMR (75 MHz, C_6_D_6_) δ: 170-105 (m, *Ph* and pyridyl), 68.1 (O–CH_2_ of *THF*), 25.8 (C–CH_2_ of *THF*). Anal. Calc. for C_50_H_60_Li_4_N_8_O_4_: C, 69.44; H, 6.99; N, 12.96%. Found: C, 69.23; H, 6.96; N, 13.13%.

### 3. Refinement

The methylene H atoms were constrained with C–H distances of 0.97Å and *U*_iso_(H) = 1.2*U*_eq_(C). The methine H atoms were constrained with C–H distances of 0.98Å and *U*_iso_(H) = 1.2*U*_eq_(C). The phenyl and pyridyl H atoms were placed in geometrically idealized positions and constrained to ride on their parent atoms, with C–H distances in the range 0.93Å and *U*_iso_(H) = 1.2*U*_eq_(C).

### Crystal data

**Table:**

[Li_4_(C_17_H_14_N_4_)_2_(C_4_H_8_O)_4_]	*Z* = 1
*M**_r_* = 864.82	*F*(000) = 460
Triclinic, *P*1	*D*_x_ = 1.161 Mg m^−^^3^
Hall symbol: -P 1	Melting point = 413–415 K
*a* = 10.3322 (10) Å	Mo *K*α radiation, λ = 0.71073 Å
*b* = 11.2231 (11) Å	Cell parameters from 2019 reflections
*c* = 12.4813 (12) Å	θ = 2.1–23.3°
α = 111.021 (2)°	µ = 0.07 mm^−^^1^
β = 105.355 (2)°	*T* = 295 K
γ = 100.763 (2)°	Prism, yellow
*V* = 1237.3 (2) Å^3^	0.20 × 0.15 × 0.15 mm

### Data collection

**Table:**

Bruker SMART CCD diffractometer	4339 independent reflections
Radiation source: fine-focus sealed tube	2180 reflections with *I* > 2σ(*I*)
Graphite monochromator	*R*_int_ = 0.036
φ and ω scans	θ_max_ = 25.0°, θ_min_ = 1.9°
Absorption correction: multi-scan (*SADABS*; Sheldrick, 1996)	*h* = −12→9
*T*_min_ = 0.986, *T*_max_ = 0.989	*k* = −13→13
6796 measured reflections	*l* = −14→14

### Refinement

**Table:**

Refinement on *F*^2^	Primary atom site location: structure-invariant direct methods
Least-squares matrix: full	Secondary atom site location: difference Fourier map
*R*[*F*^2^ > 2σ(*F*^2^)] = 0.073	Hydrogen site location: inferred from neighbouring sites
*wR*(*F*^2^) = 0.243	H-atom parameters constrained
*S* = 0.93	*w* = 1/[σ^2^(*F*_o_^2^) + (0.1525*P*)^2^] where *P* = (*F*_o_^2^ + 2*F*_c_^2^)/3
4339 reflections	(Δ/σ)_max_ < 0.001
298 parameters	Δρ_max_ = 0.37 e Å^−^^3^
61 restraints	Δρ_min_ = −0.24 e Å^−^^3^

### Special details

**Table:**

Geometry. All s.u.'s (except the s.u. in the dihedral angle between two l.s. planes) are estimated using the full covariance matrix. The cell s.u.'s are taken into account individually in the estimation of s.u.'s in distances, angles and torsion angles; correlations between s.u.'s in cell parameters are only used when they are defined by crystal symmetry. An approximate (isotropic) treatment of cell s.u.'s is used for estimating s.u.'s involving l.s. planes.
Refinement. Refinement of *F*^2^ against ALL reflections. The weighted *R*-factor *wR* and goodness of fit *S* are based on *F*^2^, conventional *R*-factors *R* are based on *F*, with *F* set to zero for negative *F*^2^. The threshold expression of *F*^2^ > σ(*F*^2^) is used only for calculating *R*-factors(gt) *etc*. and is not relevant to the choice of reflections for refinement. *R*-factors based on *F*^2^ are statistically about twice as large as those based on *F*, and *R*-factors based on ALL data will be even larger.

### Fractional atomic coordinates and isotropic or equivalent isotropic displacement parameters (Å^2^)

**Table:**

	*x*	*y*	*z*	*U*_iso_*/*U*_eq_	
Li1	0.1027 (6)	0.4551 (5)	1.0323 (5)	0.0634 (14)	
Li2	−0.1558 (7)	0.4565 (6)	0.7296 (5)	0.0719 (16)	
N1	0.1316 (3)	0.6518 (2)	1.0415 (2)	0.0507 (6)	
N2	−0.0351 (2)	0.6392 (2)	0.8668 (2)	0.0535 (7)	
N3	0.2796 (3)	0.6487 (3)	1.2128 (2)	0.0667 (8)	
N4	−0.2221 (3)	0.6071 (3)	0.7043 (3)	0.0714 (8)	
C1	0.1048 (3)	0.7175 (3)	0.9599 (3)	0.0512 (8)	
H1A	0.1048	0.8086	1.0070	0.061*	
C2	0.2139 (3)	0.7251 (3)	0.8981 (3)	0.0579 (8)	
C3	0.2904 (4)	0.8443 (4)	0.9055 (4)	0.0795 (11)	
H3	0.2775	0.9239	0.9518	0.095*	
C4	0.3849 (5)	0.8494 (6)	0.8466 (5)	0.1126 (17)	
H4	0.4342	0.9311	0.8523	0.135*	
C5	0.4057 (6)	0.7349 (8)	0.7805 (6)	0.131 (2)	
H5	0.4705	0.7379	0.7415	0.157*	
C6	0.3321 (6)	0.6141 (6)	0.7703 (5)	0.1191 (17)	
H6	0.3464	0.5356	0.7234	0.143*	
C7	0.2354 (4)	0.6077 (4)	0.8299 (4)	0.0845 (11)	
H7	0.1863	0.5256	0.8238	0.101*	
C8	0.2470 (3)	0.7188 (3)	1.1439 (3)	0.0514 (8)	
C9	0.3386 (3)	0.8526 (3)	1.1891 (3)	0.0622 (9)	
H9	0.3176	0.9039	1.1466	0.075*	
C10	0.4549 (4)	0.9057 (4)	1.2924 (3)	0.0849 (12)	
H10	0.5125	0.9932	1.3207	0.102*	
C11	0.4887 (5)	0.8315 (5)	1.3556 (4)	0.1027 (15)	
H11	0.5699	0.8661	1.4256	0.123*	
C12	0.3998 (4)	0.7060 (4)	1.3127 (4)	0.0876 (12)	
H12	0.4233	0.6554	1.3554	0.105*	
C13	−0.1040 (3)	0.6970 (3)	0.8038 (3)	0.0526 (8)	
C14	−0.0715 (4)	0.8342 (3)	0.8263 (3)	0.0633 (9)	
H14	0.0104	0.8966	0.8904	0.076*	
C15	−0.1585 (4)	0.8755 (4)	0.7552 (4)	0.0778 (11)	
H15	−0.1373	0.9661	0.7722	0.093*	
C16	−0.2783 (4)	0.7838 (5)	0.6580 (4)	0.0926 (13)	
H16	−0.3396	0.8106	0.6089	0.111*	
C17	−0.3032 (4)	0.6530 (4)	0.6367 (4)	0.0869 (13)	
H17	−0.3829	0.5906	0.5700	0.104*	
C18	0.1501 (7)	0.2098 (6)	0.8819 (7)	0.183 (3)	
H18A	0.1227	0.1720	0.9344	0.220*	
H18B	0.0681	0.1811	0.8082	0.220*	
C19	0.2581 (9)	0.1655 (7)	0.8507 (9)	0.195 (3)	
H19A	0.2663	0.0902	0.8699	0.234*	
H19B	0.2384	0.1368	0.7633	0.234*	
C20	0.3791 (8)	0.2697 (8)	0.9169 (9)	0.223 (4)	
H20A	0.4211	0.2913	0.8626	0.268*	
H20B	0.4465	0.2473	0.9710	0.268*	
C21	0.3420 (6)	0.3833 (7)	0.9886 (7)	0.180 (3)	
H21A	0.3758	0.4013	1.0749	0.216*	
H21B	0.3845	0.4630	0.9810	0.216*	
O1	0.1996 (3)	0.3504 (3)	0.9441 (3)	0.0881 (9)	
C22	−0.1386 (12)	0.1945 (8)	0.5649 (9)	0.231 (4)	
H22A	−0.2398	0.1501	0.5322	0.277*	
H22B	−0.0961	0.1731	0.6320	0.277*	
C23	−0.0822 (14)	0.1475 (9)	0.4711 (11)	0.264 (4)	
H23A	−0.0064	0.1126	0.4966	0.317*	
H23B	−0.1547	0.0782	0.3953	0.317*	
C24	−0.0334 (14)	0.2597 (12)	0.4575 (11)	0.279 (4)	
H24A	−0.0746	0.2419	0.3717	0.334*	
H24B	0.0683	0.2821	0.4797	0.334*	
C25	−0.0648 (11)	0.3669 (9)	0.5286 (8)	0.230 (4)	
H25A	0.0187	0.4455	0.5738	0.276*	
H25B	−0.1372	0.3878	0.4776	0.276*	
O2	−0.1117 (4)	0.3306 (3)	0.6075 (3)	0.1187 (12)	

### Atomic displacement parameters (Å^2^)

**Table:**

	*U*^11^	*U*^22^	*U*^33^	*U*^12^	*U*^13^	*U*^23^
Li1	0.064 (3)	0.059 (3)	0.079 (4)	0.023 (3)	0.031 (3)	0.037 (3)
Li2	0.080 (4)	0.064 (3)	0.063 (3)	0.009 (3)	0.018 (3)	0.029 (3)
N1	0.0509 (15)	0.0478 (13)	0.0523 (14)	0.0125 (12)	0.0178 (13)	0.0225 (12)
N2	0.0461 (15)	0.0511 (14)	0.0602 (15)	0.0103 (12)	0.0138 (12)	0.0272 (13)
N3	0.0630 (18)	0.0670 (17)	0.0655 (17)	0.0099 (14)	0.0115 (14)	0.0373 (15)
N4	0.0597 (18)	0.0733 (18)	0.0683 (17)	0.0037 (15)	0.0059 (15)	0.0374 (16)
C1	0.0485 (18)	0.0443 (15)	0.0595 (18)	0.0120 (13)	0.0186 (15)	0.0229 (15)
C2	0.0495 (19)	0.066 (2)	0.064 (2)	0.0160 (16)	0.0212 (16)	0.0355 (17)
C3	0.065 (2)	0.097 (3)	0.093 (3)	0.017 (2)	0.033 (2)	0.059 (2)
C4	0.085 (3)	0.151 (5)	0.135 (4)	0.024 (3)	0.056 (3)	0.093 (4)
C5	0.095 (4)	0.213 (7)	0.141 (5)	0.050 (5)	0.075 (4)	0.113 (5)
C6	0.118 (4)	0.149 (5)	0.122 (4)	0.063 (4)	0.080 (3)	0.055 (4)
C7	0.081 (3)	0.095 (3)	0.095 (3)	0.034 (2)	0.049 (2)	0.045 (2)
C8	0.0480 (18)	0.0505 (17)	0.0550 (18)	0.0119 (14)	0.0188 (15)	0.0236 (15)
C9	0.065 (2)	0.0515 (18)	0.061 (2)	0.0074 (16)	0.0144 (17)	0.0261 (16)
C10	0.078 (3)	0.072 (2)	0.074 (2)	−0.012 (2)	0.004 (2)	0.033 (2)
C11	0.082 (3)	0.108 (3)	0.082 (3)	−0.009 (3)	−0.012 (2)	0.050 (3)
C12	0.074 (3)	0.097 (3)	0.080 (3)	0.002 (2)	0.000 (2)	0.057 (2)
C13	0.0500 (18)	0.0551 (18)	0.0578 (18)	0.0145 (15)	0.0228 (15)	0.0284 (16)
C14	0.063 (2)	0.061 (2)	0.070 (2)	0.0207 (16)	0.0217 (17)	0.0331 (18)
C15	0.077 (3)	0.078 (2)	0.098 (3)	0.036 (2)	0.033 (2)	0.054 (2)
C16	0.073 (3)	0.117 (3)	0.108 (3)	0.034 (3)	0.022 (3)	0.076 (3)
C17	0.063 (3)	0.103 (3)	0.085 (3)	0.006 (2)	0.001 (2)	0.058 (2)
C18	0.167 (6)	0.094 (4)	0.239 (7)	0.018 (4)	0.127 (5)	−0.009 (4)
C19	0.182 (6)	0.104 (4)	0.262 (7)	0.062 (4)	0.092 (5)	0.020 (4)
C20	0.128 (5)	0.192 (6)	0.248 (7)	0.087 (4)	0.042 (5)	−0.015 (5)
C21	0.082 (4)	0.155 (5)	0.223 (6)	0.051 (3)	0.049 (4)	−0.006 (5)
O1	0.0756 (18)	0.0718 (17)	0.133 (2)	0.0333 (14)	0.0538 (16)	0.0442 (17)
C22	0.356 (10)	0.121 (5)	0.238 (8)	0.089 (6)	0.167 (7)	0.052 (5)
C23	0.372 (9)	0.162 (7)	0.239 (7)	0.105 (7)	0.172 (7)	0.012 (6)
C24	0.365 (9)	0.240 (9)	0.225 (7)	0.080 (8)	0.197 (6)	0.037 (7)
C25	0.350 (9)	0.202 (8)	0.191 (6)	0.073 (7)	0.195 (6)	0.078 (6)
O2	0.172 (3)	0.085 (2)	0.091 (2)	0.021 (2)	0.070 (2)	0.0226 (17)

### Geometric parameters (Å, º)

**Table:**

Li1—O1	1.921 (6)	C9—C10	1.350 (5)
Li1—N2^i^	2.084 (6)	C9—H9	0.9300
Li1—N1	2.128 (6)	C10—C11	1.368 (5)
Li1—N1^i^	2.256 (6)	C10—H10	0.9300
Li1—N3	2.441 (6)	C11—C12	1.352 (5)
Li1—Li1^i^	2.578 (10)	C11—H11	0.9300
Li1—Li2^i^	2.633 (8)	C12—Li2^i^	2.617 (7)
Li1—C1^i^	2.656 (6)	C12—H12	0.9300
Li1—C8	2.686 (6)	C13—C14	1.418 (4)
Li2—O2	1.895 (7)	C14—C15	1.356 (5)
Li2—N3^i^	2.020 (7)	C14—H14	0.9300
Li2—N2	2.022 (6)	C15—C16	1.377 (5)
Li2—N4	2.032 (7)	C15—H15	0.9300
Li2—C13	2.415 (6)	C16—C17	1.354 (5)
Li2—C12^i^	2.617 (7)	C16—H16	0.9300
Li2—Li1^i^	2.633 (8)	C17—H17	0.9300
N1—C8	1.337 (4)	C18—C19	1.393 (9)
N1—C1	1.458 (4)	C18—O1	1.402 (6)
N1—Li1^i^	2.256 (6)	C18—H18A	0.9700
N2—C13	1.336 (4)	C18—H18B	0.9700
N2—C1	1.455 (4)	C19—C20	1.352 (9)
N2—Li1^i^	2.084 (6)	C19—H19A	0.9700
N3—C12	1.349 (4)	C19—H19B	0.9700
N3—C8	1.379 (4)	C20—C21	1.455 (8)
N3—Li2^i^	2.020 (7)	C20—H20A	0.9700
N4—C17	1.333 (4)	C20—H20B	0.9700
N4—C13	1.374 (4)	C21—O1	1.351 (5)
C1—C2	1.531 (4)	C21—H21A	0.9700
C1—Li1^i^	2.656 (6)	C21—H21B	0.9700
C1—H1A	0.9800	C22—O2	1.369 (8)
C2—C3	1.378 (5)	C22—C23	1.422 (12)
C2—C7	1.384 (5)	C22—H22A	0.9700
C3—C4	1.374 (6)	C22—H22B	0.9700
C3—H3	0.9300	C23—C24	1.352 (11)
C4—C5	1.347 (8)	C23—H23A	0.9700
C4—H4	0.9300	C23—H23B	0.9700
C5—C6	1.369 (8)	C24—C25	1.368 (11)
C5—H5	0.9300	C24—H24A	0.9700
C6—C7	1.400 (6)	C24—H24B	0.9700
C6—H6	0.9300	C25—O2	1.355 (7)
C7—H7	0.9300	C25—H25A	0.9700
C8—C9	1.430 (4)	C25—H25B	0.9700
			
O1—Li1—N2^i^	107.8 (3)	C5—C6—C7	120.5 (5)
O1—Li1—N1	116.0 (3)	C5—C6—H6	119.7
N2^i^—Li1—N1	134.1 (3)	C7—C6—H6	119.7
O1—Li1—N1^i^	112.1 (3)	C2—C7—C6	119.1 (4)
N2^i^—Li1—N1^i^	64.89 (18)	C2—C7—H7	120.5
N1—Li1—N1^i^	108.0 (2)	C6—C7—H7	120.5
O1—Li1—N3	108.2 (3)	N1—C8—N3	115.0 (3)
N2^i^—Li1—N3	94.7 (2)	N1—C8—C9	127.9 (3)
N1—Li1—N3	59.72 (16)	N3—C8—C9	117.2 (3)
N1^i^—Li1—N3	138.7 (3)	N1—C8—Li1	51.44 (18)
O1—Li1—Li1^i^	133.7 (4)	N3—C8—Li1	64.8 (2)
N2^i^—Li1—Li1^i^	101.7 (3)	C9—C8—Li1	169.4 (3)
N1—Li1—Li1^i^	56.3 (2)	C10—C9—C8	121.2 (3)
N1^i^—Li1—Li1^i^	51.7 (2)	C10—C9—H9	119.4
N3—Li1—Li1^i^	104.0 (3)	C8—C9—H9	119.4
O1—Li1—Li2^i^	125.5 (3)	C9—C10—C11	120.4 (3)
N2^i^—Li1—Li2^i^	49.08 (19)	C9—C10—H10	119.8
N1—Li1—Li2^i^	92.4 (2)	C11—C10—H10	119.8
N1^i^—Li1—Li2^i^	100.1 (3)	C12—C11—C10	117.6 (4)
N3—Li1—Li2^i^	46.73 (17)	C12—C11—H11	121.2
Li1^i^—Li1—Li2^i^	100.8 (3)	C10—C11—H11	121.2
O1—Li1—C1^i^	106.6 (2)	N3—C12—C11	124.9 (4)
N2^i^—Li1—C1^i^	33.02 (12)	N3—C12—Li2^i^	49.5 (2)
N1—Li1—C1^i^	133.0 (3)	C11—C12—Li2^i^	150.8 (4)
N1^i^—Li1—C1^i^	33.27 (11)	N3—C12—H12	117.5
N3—Li1—C1^i^	124.3 (2)	C11—C12—H12	117.5
Li1^i^—Li1—C1^i^	80.3 (3)	Li2^i^—C12—H12	76.1
Li2^i^—Li1—C1^i^	77.7 (2)	N2—C13—N4	113.1 (3)
O1—Li1—C8	111.9 (3)	N2—C13—C14	129.1 (3)
N2^i^—Li1—C8	119.9 (3)	N4—C13—C14	117.8 (3)
N1—Li1—C8	29.44 (11)	N2—C13—Li2	56.8 (2)
N1^i^—Li1—C8	130.5 (2)	N4—C13—Li2	57.2 (2)
N3—Li1—C8	30.74 (11)	C14—C13—Li2	169.5 (3)
Li1^i^—Li1—C8	81.4 (2)	C15—C14—C13	120.7 (3)
Li2^i^—Li1—C8	71.15 (19)	C15—C14—H14	119.7
C1^i^—Li1—C8	139.8 (2)	C13—C14—H14	119.7
O2—Li2—N3^i^	107.2 (3)	C14—C15—C16	120.3 (4)
O2—Li2—N2	130.7 (4)	C14—C15—H15	119.9
N3^i^—Li2—N2	111.3 (3)	C16—C15—H15	119.9
O2—Li2—N4	121.6 (3)	C17—C16—C15	117.3 (3)
N3^i^—Li2—N4	113.6 (3)	C17—C16—H16	121.3
N2—Li2—N4	67.9 (2)	C15—C16—H16	121.3
O2—Li2—C13	131.3 (3)	N4—C17—C16	124.9 (4)
N3^i^—Li2—C13	121.3 (3)	N4—C17—H17	117.5
N2—Li2—C13	33.59 (13)	C16—C17—H17	117.5
N4—Li2—C13	34.67 (14)	C19—C18—O1	108.4 (5)
O2—Li2—C12^i^	95.7 (3)	C19—C18—H18A	110.0
N3^i^—Li2—C12^i^	30.49 (14)	O1—C18—H18A	110.0
N2—Li2—C12^i^	132.5 (3)	C19—C18—H18B	110.0
N4—Li2—C12^i^	98.8 (3)	O1—C18—H18B	110.0
C13—Li2—C12^i^	123.6 (3)	H18A—C18—H18B	108.4
O2—Li2—Li1^i^	138.0 (3)	C20—C19—C18	107.3 (6)
N3^i^—Li2—Li1^i^	61.6 (2)	C20—C19—H19A	110.3
N2—Li2—Li1^i^	51.15 (18)	C18—C19—H19A	110.3
N4—Li2—Li1^i^	98.5 (3)	C20—C19—H19B	110.3
C13—Li2—Li1^i^	75.6 (2)	C18—C19—H19B	110.3
C12^i^—Li2—Li1^i^	89.3 (2)	H19A—C19—H19B	108.5
C8—N1—C1	115.8 (2)	C19—C20—C21	106.9 (6)
C8—N1—Li1	99.1 (2)	C19—C20—H20A	110.3
C1—N1—Li1	139.7 (2)	C21—C20—H20A	110.3
C8—N1—Li1^i^	144.3 (2)	C19—C20—H20B	110.3
C1—N1—Li1^i^	88.6 (2)	C21—C20—H20B	110.3
Li1—N1—Li1^i^	72.0 (2)	H20A—C20—H20B	108.6
C13—N2—C1	118.7 (2)	O1—C21—C20	107.3 (5)
C13—N2—Li2	89.6 (2)	O1—C21—H21A	110.3
C1—N2—Li2	144.8 (3)	C20—C21—H21A	110.3
C13—N2—Li1^i^	128.5 (2)	O1—C21—H21B	110.3
C1—N2—Li1^i^	95.7 (2)	C20—C21—H21B	110.3
Li2—N2—Li1^i^	79.8 (2)	H21A—C21—H21B	108.5
C12—N3—C8	118.5 (3)	C21—O1—C18	105.4 (4)
C12—N3—Li2^i^	100.0 (3)	C21—O1—Li1	121.2 (4)
C8—N3—Li2^i^	130.2 (3)	C18—O1—Li1	123.5 (3)
C12—N3—Li1	152.4 (3)	O2—C22—C23	109.8 (8)
C8—N3—Li1	84.5 (2)	O2—C22—H22A	109.7
Li2^i^—N3—Li1	71.6 (2)	C23—C22—H22A	109.7
C17—N4—C13	118.9 (3)	O2—C22—H22B	109.7
C17—N4—Li2	151.9 (3)	C23—C22—H22B	109.7
C13—N4—Li2	88.1 (2)	H22A—C22—H22B	108.2
N2—C1—N1	106.5 (2)	C24—C23—C22	102.6 (9)
N2—C1—C2	109.9 (2)	C24—C23—H23A	111.2
N1—C1—C2	112.9 (2)	C22—C23—H23A	111.2
N2—C1—Li1^i^	51.31 (18)	C24—C23—H23B	111.2
N1—C1—Li1^i^	58.11 (18)	C22—C23—H23B	111.2
C2—C1—Li1^i^	142.4 (2)	H23A—C23—H23B	109.2
N2—C1—H1A	109.2	C23—C24—C25	112.4 (10)
N1—C1—H1A	109.2	C23—C24—H24A	109.1
C2—C1—H1A	109.2	C25—C24—H24A	109.1
Li1^i^—C1—H1A	108.0	C23—C24—H24B	109.1
C3—C2—C7	118.2 (3)	C25—C24—H24B	109.1
C3—C2—C1	122.7 (3)	H24A—C24—H24B	107.9
C7—C2—C1	119.1 (3)	O2—C25—C24	106.5 (8)
C4—C3—C2	122.2 (4)	O2—C25—H25A	110.4
C4—C3—H3	118.9	C24—C25—H25A	110.4
C2—C3—H3	118.9	O2—C25—H25B	110.4
C5—C4—C3	119.3 (5)	C24—C25—H25B	110.4
C5—C4—H4	120.3	H25A—C25—H25B	108.6
C3—C4—H4	120.3	C25—O2—C22	106.9 (6)
C4—C5—C6	120.6 (5)	C25—O2—Li2	119.8 (5)
C4—C5—H5	119.7	C22—O2—Li2	132.3 (5)
C6—C5—H5	119.7		

Symmetry code: (i) −*x*, −*y*+1, −*z*+2.
